# Dating app usage and motivations for dating app usage are associated with increased disordered eating

**DOI:** 10.1186/s40337-022-00693-9

**Published:** 2022-11-28

**Authors:** K. Blake, J. Portingale, S. Giles, S. Griffiths, I. Krug

**Affiliations:** 1 Melbourne School of Psychological Sciences, University of Melbourne, Redomond Barry Builiding, Level 7, Room 707, Melbourne, VIC 3010 Australia; 2 School of Biological, Earth, and Environmental Sciences, UNSW Sydney, Sydney, NSW 2052 Australia

**Keywords:** Dating apps, Disordered eating, Emotion regulation, Rejection sensitivity, Social rank

## Abstract

The centrality of physical appearance in dating app environments may constitute an appearance-related pressure that increases the likelihood of body dissatisfaction (BD) and disordered eating (DE), thus exacerbating the relationship between DE-predictive traits and DE itself. Although dating app use has been linked to BD and DE, prior research has also neglected the role of individuals’ dating app use motivations and relevant traits in eating pathology. To address these gaps, the current study investigated whether dating app usage moderated the effects of appearance-based rejection sensitivity, fear of negative evaluation, emotion dysregulation, and perceived social rank on DE. We also examined the unique effects of individuals’ dating app use motivations on DE. Participants (*N* = 690) completed baseline measures of demographic and trait variables including dating app usage. DE was positively associated with female gender, higher body mass index, a history of eating disorder (ED) diagnosis, appearance-based rejection sensitivity, and emotion dysregulation. There was a small, positive association between dating app usage and DE, indicating that dating app users were more likely to report DE symptoms, appearance-based rejection sensitivity, and emotion dysregulation. No investigated predictor was moderated by dating app usage, but four of the six measured motivations for using dating apps (love, self-worth, ease of communication, and thrill of excitement motivations) were associated with DE among the dating app user sample (casual sex and trendiness motivations were not). Given that DE behaviours can lead to EDs, the present findings suggest that lifetime dating app usage may increase socio-cultural appearance pressures that confer risk for DE.

## Plain English summary

Existing research evidence, although scant, has linked dating app use to body dissatisfaction (BD) and disordered eating (DE). Yet, little is known about the relationship between individuals’ dating app use motivations and relevant traits in eating pathology. To address these gaps, the current study investigated whether dating app usage moderated the effects of appearance-based rejection sensitivity (appearance-RS), fear of negative evaluation (FNE), emotion dysregulation, and perceived social rank on DE, and the unique association between individuals’ dating app use motivations and DE. Participants (*N* = 690) completed baseline measures of demographic and trait variables including dating app usage. We found that dating app users were more likely to engage in DE behaviours, appearance-RS, and emotion dysregulation than non-users. Furthermore, four of the six measured motivations for using dating apps were associated with DE among the dating app user sample. We conclude that lifetime dating app usage may constitute a socio-cultural appearance pressure which confers DE risk. Our results showed also novel insights into the varied motivations for dating app use and their impacts on DE. As dating app use continues to proliferate, enhancing our understanding of how, why and for whom it may be harmful remains a salient area of research.

## Introduction

Modern dating is increasingly conducted through online dating applications (apps) such as Tinder, Grindr and Bumble, which use geolocation and profile pictures to pair potential dating partners. Popular “swiping-based” dating apps such as Tinder, Grindr and Bumble, enable users to quickly appraise a large number of potential “matches” by viewing others’ profiles and rapidly indicating their interest (e.g., by “swiping left” or “swiping right”). With the average Tinder user swiping left or right on 140 people per day [35] and little information available except the users’ profile picture, appraisals are often based primarily on the users’ physical attractiveness. “Non-swiping based’ dating apps such as Hinge encourage a more detailed filtering process based on non-appearance-based features such as biographies; however, nonetheless, still foreground profile pictures, and thus, appearance-centred evaluations. Given the central role of physical appearance concerns in exacerbating body dissatisfaction (BD; [11]), here, we investigate whether and why dating app usage increases the risk of sub-clinical disordered eating (DE). To do so, we examined associations between dating app usage, DE, and a range of trait-level and psychological predictors that are known to exacerbate DE. More specifically, we assessed whether the effects of trait-level and psychological predictors on DE were stronger among dating app users compared to non-users, and further, determined whether specific motivations for dating app usage exacerbated DE.

### Online dating and eating disorders

Dating apps have become increasingly popular, with an estimated 207 million people currently using dating apps worldwide [9]. Although physical appearance is commonly important for dating [3], it is especially pertinent for online dating, which commonly involves rapid judgements based on potential dates’ profile pictures [14]. The centrality of physical appearance in these judgements can result in users’ placing a disproportionate degree of importance on physical attractiveness, with negative flow-on effects on self-esteem and body satisfaction [37]. Given that preoccupations with shape and weight are core psychopathological features in Fairburn’s transdiagnostic model for eating disorders (EDs [11], and that dating app users are more likely to engage in unhealthy weight control behaviours than non-users [41], there are good reasons to suspect that dating app usage may foster BD and DE.

To date, only two published studies have examined the effects of dating app usage on DE [30, 41], and three additional studies have examined the effects of dating app usage on BD among predominately heterosexual populations [12, 33, 37]. In a comparison of 100 Tinder users and 847 non-users, Strubel and Petrie [37] found positive associations between dating app use and thin-ideal internalization, appearance comparisons, body shame, body surveillance and BD. In a sample of 170 dating app users, Rodgers et al. [33] found a positive association between frequent dating app use and body shame among men but not women, and a positive association between dating app use and BD among women experiencing negative affect after using dating apps. In a recent male sample of 179 app users and 480 non-users, Fong et al. [12] found positive associations between dating app use and body image disturbance, muscularity dissatisfaction, height dissatisfaction, and overall dissatisfaction. In a sample comprised of 392 app users and 1334 non-users, Tran et al. [41] found that dating app users engaged in more self-induced vomiting, fasting, and diet pill use compared to non-users. Moreover, in a recently published ecological momentary assessment study of 296 females (32% of lifetime dating app users), Portingale et al. [30] found a positive association between lifetime dating app use and everyday urges for binge-eating/purging.

Collectively, this small body of research suggests that dating app use is associated with BD and DE, and related symptoms. One key limitation in these studies, however, is that no study has examined individuals’ motivations to use online dating apps and whether these motivations affect eating pathology. Emerging research indicates that individuals use apps for varying reasons that differ by age and gender [39], and some of these motivations—such as self-worth validation—are relevant to the development of ED symptomology [10]. Furthermore, a systematic review of 20 studies on the relationship between social networking site usage, body image concerns, and disordered eating found a positive link between these factors [18]. Viewing and uploading images, as well as seeking negative criticism through status updates, were found to be particularly troublesome. According to the different motivations for dating app usage outlined by Sumter et al. [39], viewing and publishing photographs could be related to enhancing self-worth, whereas seeking feedback via status updates could be considered a form of communication. Hence, the motivations outlined by Sumter et al. [39] also seem to have relevance in the relationship between dating apps and disordered eating symptoms. An examination of these motivational factors would help understand the individual characteristics that might be linked to DE among dating app users versus non-users. It could also provide insights into the functional role of DE which develops in response to dating app use.

A second limitation is the failure to control for important trait-level and psychological predictors known to exacerbate DE, especially those which are salient in a dating app environment. These predictors include appearance-based rejection sensitivity [20, 24], fear of negative evaluation (FNE) [16], social rank [6, 27] and emotion dysregulation [34]. As we explain below, dating app usage could potentially increase their detrimental effects.

### Appearance-based rejection sensitivity and eating disorders

Appearance-based rejection sensitivity is a socio-affective processing bias where individuals are especially conscious of rejection based on their physical appearance [29]. Appearance-based rejection sensitivity is positively associated with BD [5] and DE [29] in community samples. Given the centrality of physical appearance within dating apps, individuals sensitive to appearance-based rejection may be especially likely to attribute dating app rejection to their physical appearance [30]. This, in turn, might intensify DE symptoms among app-users [30].

### Social rank and eating disorders

Social ranking theory suggests that social rank plays a significant role in social interactions in many group-living species, such that mood and social behaviours are influenced by perceptions of one’s social rank/status [31]. Overall, studies have shown that individuals with EDs perceive that they have low social rank and are inferior to others; they also tend to display submissive behaviours [6, 43]. People with low perceived social rank may employ DE in an attempt to elevate their rank by achieving thin or muscular body ideals. These individuals may be more acutely affected by the appearance-centric dating app environment and thus engage in DE to avoid any unwanted inferiority associated with being overlooked or rejected.

### Fear of negative evaluation and eating disorders

FNE is a component of social anxiety disorder and refers to anxiety about being negatively evaluated [16]. Models of FNE suggest this process also operates within a psycho-evolutionary framework, as FNE may function to avoid downward shifts in social rank. It is also a salient feature of EDs and is associated with greater BD [26] and a higher odds of meeting diagnostic criteria for any ED [42]. Anxiety about how others evaluate one's body weight and shape is particularly relevant in the context of dating apps, in which judgements of others are often based on physical appearance. For this reason, fear of negative evaluation may predispose individuals—especially dating app users—to engage in DE to reduce their likelihood of being negatively evaluated by others.

### Emotion dysregulation and eating disorders

Emotion dysregulation refers to the phenomenon where people are unable to effectively modulate and respond to an emotional experience [15]. Emotion dysregulation is a common feature of clinical and sub-clinical DE [34] and is implicated as a core maintaining factor in the transdiagnostic model of EDs [11]. Emotion dysregulation is thus a significant factor in the development and maintenance of BD and DE. Dating app use is associated with psychological distress, the impacts of which may be particularly unfavourable for individuals with pre-existing difficulties in modulating affective experiences, which may then lead to bingeing or restriction, to regulate emotional states. For this reason, we examined whether emotion dysregulation was associated with DE among dating app users and non-users.

### The present study

The centrality of physical appearance in dating app environments may constitute an appearance-related pressure that increases the likelihood of presenting with BD and DE. Psychological predictors including sensitivity to appearance-based rejection, fear of negative evaluation, emotion dysregulation, and perceived lowered social rank may contribute to DE differentially among dating app users compared to non-users. We aimed to investigate whether dating app usage moderated the relationship of these variables on DE. Given some motivations for using dating apps are also associated with DE (e.g., [10]), we further aimed to assess whether individuals motivations for using the apps augmented or attenuated DE. Investigating the relationship between dating app usage, the motivations to engage in their usage, and the above-outlined variables associated with DEs may foster an understanding of the individual differences driving DE. Such findings may also provide potential prevention efforts to improve body image and eating patterns in dating app users.

## Method

### Participants

A total of 690 participants (532 women; age *M* = 20.30 years, *SD* = 4.51, range = 17–51) took part in the current study, of whom 310 individuals had used dating apps previously. Approximately 11% self-reported currently or previously suffering from an ED and the body mass index (BMI) ranged from 15.35 to 66.06 kg/m^2^ (*M* = 22.11, *SD* = 4.33). As shown in Table 1, the sample was ethnically diverse: 41.2% were White, 30.7% were Eastern Asian, 15.6% were Southern or Southeast Asian, and the remainder were Hispanic or Latino American (0.3%), Middle Eastern (2.9%), or Other (9.3%). Most of the sample was heterosexual (86.8%), 8.1% were bisexual, and the remainder were homosexual (3.3%) or reported their sexuality as other (1.3%). Three-quarters (76.3%) of the sample had completed up to a Year 12 education, 51.8% were unemployed and 95.1% had never been married. English (66.6%) was the most common language spoken.

**Table 1:** Sociodemographic characteristics for the total sample, and by dating app user status

Demographic variable	Statistics				
	Dating app user (*n* = 310, 45.1%)	Dating app non-user (*n* = 377, 54.9%)	Total (*N* = 687)	*t*/χ^2^	*p*
Age (*M* ± *SD*)	20.99 ± 4.92	22.30 ± 8.47	20.28 ± 4.63		
BMI (*M* ± *SD*)	22.47 ± 3.66	22.01 ± 3.73	22.11 ± 4.33		
*Gender (n, %)*				10.06	**0.007**
Female	222 (71.6%)	307 (81.4%)	529 (77%)		
Male	87 (28.1%)	70 (18.6%)	157 (22.9%)		
Other	1 (0.3%)	0 (0%)	1 (0.1%)		
*Ethnicity (n, %)*				24.01	** <0.001**
White	152 (49%)	131 (34.7%)	285 (41.2%)		
Eastern Asian	71 (22.9%)	140 (37.1%)	212 (30.7%)		
Southern Asian/Southeast Asian	48 (15.5%)	59 (15.6%)	108 (15.6%)		
Other	39 (12.6%)	47 (12.5%)	86 (12.5%)		
*Highest education completed (n, %)*				19.96	**0.006**
Year 12 or below	220 (71%)	305 (80.9%)	525 (76.3%)		
Certificate/diploma	19 (6.1%)	23 (6.1%)	42 (6.1%)		
Bachelor’s degree	45 (14.5%)	36 (9.5%)	82 (11.9%)		
Postgraduate degree	26 (8%)	13 (3.4%)	39 (5.7%)		
*Current paid employment status (n, %)*				13.16	** <0.001**
Employed	173 (55.8%)	158 (41.9%)	331 (48.2%)		
Not employed	137 (44.2%)	219 (58.1%)	356 (51.8%)		
*Main language spoken at home (n, %)*				18.94	** <0.001**
English	233 (75.2%)	224 (59.4%)	458 (66.6%)		
Other	77 (24.8%)	153 (40.6%)	230 (33.4%)		
*Sexual orientation (n, %)*				13.97	**0.003**
Heterosexual	255 (82.3%)	343 (91%)	599 (87.1%)		
Homosexual	16 (5.2%)	7 (1.9%)	23 (3.3%)		
Bisexual	35 (11.3%)	21 (5.6%)	56 (8.1%)		
Other	4 (1.3%)	6 (1.6%)	10 (1.5%)		
*Marital status (n, %)*				5.43	0.246
Married	3 (0.9%)	6 (1.6%)	9 (1.3%)		
De facto	13 (4.2%)	8 (2.1%)	21 (3.1%)		
Separated	2 (0.6%)	1 (0.2%)	2 (0.3%)		
Divorced	1 (0.3%)	1 (0.2%)	2 (0.3%)		
Never married	291 (93.9%)	361 (95.8%)	654 (95.1%)		
*History of an eating disorder*					
Yes	42 (14.5%)	31 (8.5%)	73 (11.1%)	5.92	**0.017**
No	248 (85.5%)	335 (91.5%)	583 (88.9%)		

Significant *p* values bolded

*BMI* Body Mass Index (kg/m^2^), *M* mean, *SD* standard deviation

t-test for continuous variables, chi-squared test for categorical variables

### Procedure

Participants aged over 18 years were recruited from several sources within the University of [redacted], social media advertisements by the authors, and research listservs. Participants completed a 30-min online survey asking about the demographics, lifetime dating app usage and the above-outlined measures. If participants answered that they engaged in dating app usage throughout their lifetime, they were also asked questions about their dating app usage frequency and motivations for using dating apps. As compensation for their time, participants from the university's research experience program (REP) were provided one unit of course credit, whilst those from the community were entered into a draw to win one of three $100 (AUD) iTunes gift cards. The study was approved by the University of [*redacted for peer-review*] Human Research and Ethics Committee and all participants provided electronically written informed consent to participate.

### Measures

#### Demographics and ED diagnoses

The baseline questionnaire obtained information concerning age, gender, ethnic background, sexual orientation, marital status, and the highest level of education completed. Current height in centimetres and weight in kilograms was also self-reported, allowing us to calculate individuals’ BMIs. Finally, participants were also asked whether they have ever experienced an ED.

#### Dating app variables

Dating app usage was assessed by asking whether participants had ever used a dating app (45% yes). We then evaluated users’ motivation for using dating apps by asking whether they used apps for Casual Sex, Ease of Communication, Self-Worth Validation, Thrill of Excitement, Trendiness, or Love. These categories for motivation for app use were taken from Sumter et al. [39] Participants were able to indicate all the motivations that applied to them. To measure current app use frequency, we also asked how many swipes users engaged in per week (*M* = 55, *SD* = 91.11, range = 0–1000).

#### Appearance-based rejection sensitivity

Appearance-based rejection sensitivity was measured via the Appearance-based Rejection Sensitivity Scale short-form scale [28]. The scale presented 10 hypothetical scenarios (e.g., *“You are leaving your house to go on a first date when you notice a blemish on your face*”) for which the participant indicated their anxiety (1 = *very unconcerned, 6* = *very concerned),* and expectation of rejection (1 = *very unlikely,* 6 = *very likely*). For each scenario, anxiety scores were multiplied by rejection scores, which were then averaged across scenarios; leading to a mean score with a range of 1–36 *(M* = 14.72, *SD* = 6.78, Cronbach’s α = 0.90). Higher scores indicate higher appearance-based sensitivity to rejection.

#### Social rank

Social rank was measured using the 11-item Social Comparison Scale [1]. Participants made a global social comparison of themselves in relation to others with a series of bipolar constructs rated 1–10; 1—low subjective social ranking, 10—high subjective social ranking (e.g., *“In relation to others I feel….*”: 1 = *inferior,* 10 = *superior*). Items were summed, with high scores indicating feelings of superiority and self-perceived high rank (*M* = 64.28, *SD* = 15.05, α = 0.91).

#### Fear of negative evaluation

We used the 12-item Brief Fear of Negative Evaluation Scale [23] to measure fear of being negatively evaluated. Each item (e.g., “*I am afraid that others will not approve of me*”) was rated on a 5-point Likert scale (1 = *not at all,* 5 = *extremely*) with a range of 12–60. Items were summed, with higher scores indicating greater fear of negative evaluation (*M* = 39.02, *SD* = 9.18, α = 0.76).

#### Emotion dysregulation

Emotion dysregulation was measured using the 18-item Brief Version of the Difficulties in Emotion Regulation Scale [19]. Items (e.g.,”*I pay attention to how I feel*”) were rated on a 5-point Likert scale (1 = *almost never,* 5 = *almost always*) and summed to create a total score (range = 18–90); higher scores represented more difficulty regulating emotions (*M* = 50.97, *SD* = 12.36, α = 0.89).

#### Disordered eating

We evaluated DE using the 12-item Eating Disorder Examination Questionnaire Short Form [13], which assesses DE symptoms over the last 7 days. Items (e.g.,”*Have you had a definite fear that you might gain weight?*") were rated from zero (*0 days/Not at all*) to three (*6–7 days/Markedly*), and then summed, with higher scores indicating elevated DE psychopathology (*M* = 9.79, *SD* = 7.20, α = 0.89).

### Data analysis

We were interested in the magnitude of effects of dating app variables on DE, controlling for known predictors (appearance-based rejection sensitivity, social rank, fear of negative evaluation, emotion dysregulation, ED diagnosis) of this outcome variable. Using multiple linear regression, in Model 1 we tested trait-level sociodemographic and clinical predictors (gender, BMI, ED history; Step 1), psychological predictors (appearance-based rejection sensitivity, social rank, fear of negative evaluation, emotion dysregulation; Step 2), and dating app usage (Step 3) on DE for the full sample of app-users and non-app users. We chose these trait level covariates because they have been implicated as correlates or risk factors for disordered eating (i.e., female gender, BMI, and history of an ED). In Model 2 we examined whether dating app usage moderated the effects of trait and psychological predictors on DE. In Model 3 we examined the effects of these same trait predictors (Step 1) and psychological predictors (Step 2) on DE for app-users only; in this model, we also entered the six motivators for dating app usage (Step 3). We examined collinearity using variance inflation factors: all variance inflation factors were below 2.0, indicating that problematic levels of multi-collinearity were unlikely to be present. Where variables were summed, no missing data were present among participants (i.e., all summed scores were accurate, and not biased by missing data on some items within scales). To minimise the deletion of available data, any other missing data were treated using pairwise deletion. A power analysis for linear regression with 15 predictors (Model 2) indicated that the achieved power to detect a medium effect (*f* = 0.39) for our recruited sample of 690 individuals was *b* = 0.996.

## Results

Table 1 shows the differences between dating app users and non-users on sociodemographic variables. App users and non-app users differed in a variety of ways, including gender, ethnicity, education, employment status, and sexual orientation. Among the app using sample, the most commonly used dating app was Tinder (40%), followed by Bumble (6.1%), OKCupid (4.1%), Grindr (2.3%), Her (1.6%), and Coffee Meets Bagel (0.7%). Thrill of Excitement was the most frequent motivation people noted for dating app usage (22.9%), followed by Trendiness (15.8%), Ease of Communication (15.5%); Self-Worth Validation (14.3%), Love (13.6%), and Casual Sex (10.6%). We then examined differences between app users and non-users on the variables included in our regression models. No significant group differences were found, except for DE, which was higher for the dating app user group compared to the non-dating app user group (see Table 2).

**Table 2:** Differences between dating app users and non-users on trait variables included in the regression models

Trait variables	Statistics			
	Dating app user (*n* = 310; *M* ± *SD*)	Dating app non-user (*n* = 377; *M* ± *SD*)	*t*	*p*
Appearance-based rejection sensitivity	15.06 ± 7.04	14.43 ± 6.56	1.188	0.235
Social rank	64.87 ± 16.08	63.82 ± 14.17	0.886	0.376
Fear of negative evaluation	38.97 ± 9.20	39.04 ± 9.18	− 0.095	0.924
Emotion dysregulation	46.78 ± 13.16	46.31 ± 12.21	0.478	0.633
Disordered eating	10.43 ± 7.50	9.25 ± 6.91	2.104	**0.036**

Significant *p* values are bolded

*M* mean, *SD* standard deviation

Table 3 shows the correlation between dependent variables and Table 4 shows the effect of trait, psychological, and dating-related variables on DE (Model 1). DE was positively associated with female gender, higher BMI, a history of an ED, appearance-based rejection sensitivity, and emotion dysregulation. The effects of social rank and fear of negative evaluation on DE did not reach statistical significance. There was a small, positive association between dating app usage and DE, indicating that dating app users were more likely to report DE symptoms. In Model 2 we entered moderation terms between dating app usage and each of the sociodemographic, clinical, and psychological predictors that were associated with DE in Model 1. No trait-level or psychological predictor was moderated by dating app usage (Wald’s χ^2^s range: 0.001–1.92, *p*s range: 0.166–0.976). Thus, the effects of trait and psychological-level predictors on DE did not differ by dating app usage sub-groups.

**Table 3:** Pearson correlations among dating app use and trait variables included in the regression models

		Dating app use	Fear of negative evaluation	Appearance-based rejection sensitivity	Social rank	Disordered eating
Correlations	Fear of negative evaluation	− 0.002				
	Appearance-based rejection sensitivity	0.048	0.525**			
	Social rank	0.034	− 0.439**	− 0.385**		
	Disordered eating	0.084*	0.326**	0.474**	− 0.284**	
	Emotion dysregulation	0.022	0.424**	0.403**	− 0.342**	0.355**

*Correlation is significant at the 0.05 level (2-tailed)

**Correlation is significant at the 0.01 level (2-tailed)

**Table 4:** Effects of trait, demographic, emotion dysregulation, and dating app variables on disordered eating, for the whole sample (Model 1) and dating app users only (Model 3)

	Model 1				Model 3		
	*β*	*t*	*p*	*Β*		*t*	*p*
*Step 1*							
Female gender	0.19	5.13	** <0.001**	0.19		3.40	**0.001**
BMI	0.17	4.48	** <0.001**	0.15		2.62	**0.009**
History of an eating disorder	0.21	5.49	** <0.001**	0.21		3.75	** <0.001**
*Step 2*							
Appearance-based rejection sensitivity	0.32	7.80	** <0.001**	0.36		6.15	** <0.001**
Social rank	− 0.05	− 1.41	0.159	− 0.01		− 0.13	0.895
Fear of negative evaluation	0.05	1.20	0.231	0.06		0.94	0.349
Emotion dysregulation	0.15	4.01	** <0.001**	0.21		3.85	** <0.001**
*Step 3*							
Dating app user	0.07	2.15	**0.032**				
Using apps for love				− 0.12		− 2.42	**0.016**
Using apps for casual sex				0.07		1.43	0.155
Using apps for ease of communication				0.12		2.59	**0.010**
Using apps for self-worth validation				0.13		2.50	**0.013**
Using apps for thrill of excitement				− 0.10		− 2.11	**0.036**
Using apps for trendiness				− 0.05		− 1.03	0.306

Model 1: Step 1 *F*(3, 651) = 28.31, *p* < .001, *R*^2^ = .12; Step 2 *F*(7, 651) = 42.02, *p* < .001, *R*^2^ = .31; Step 3 *F*(8, 651) = 37.55, *p* < .001, *R*^2^ = .32. Model 3 Step 1 *F*(3, 288) = 13.49, *p* < .001, *R*^2^ = .12; Step 2 *F*(7, 288) = 24.00, *p* < .001, *R*^2^ = .37. Step 3 *F*(13, 275) = 15.28, *p* < .001, *R*^2^ = .42

Significant *p* values were bolded

As shown in Model 3 (Table 4), four of the six motivations for using dating apps were associated with DE among the dating app user sample. Using dating apps for love and the thrill of excitement was negatively associated with DE, whereas using dating apps for ease of communication or validation of self-worth was positively associated with DE. The effects of using dating apps for casual sex or trendiness on DE were not statistically significant.

## Discussion

The popularity of dating apps has grown enormously in recent years, with a systematic review of 70 studies suggesting that dating app participation prevalence among young adults reaches 40–50% [8]. In a sample of 690 people, we sought to investigate the associations between known correlates of EDs, dating app use and sub-clinical DE. We were specifically interested in whether the relationship between known correlates of EDs and sub-clinical DE was stronger among app users compared to non-app users. Consistent with past work, DE was more prevalent among people who were women, had a higher BMI, had a history of an ED, had trouble regulating their emotions, or were especially sensitive to appearance-related rejection [5, 29, 34, 41]. We also found that dating app users were more likely to engage in DE behaviours than non-app users, but note that the effect size was small. Effects of these predictors were not moderated by dating app usage: In other words, trait-level and psychological predictors did not exert stronger effects among the dating app user sample. Within the dating app user sample, however, certain motivations for using apps were associated with a higher likelihood of reporting DE.

Consistent with prior research [30, 41], lifetime dating app users were more likely to engage in DE than non-app users. Importantly, the current findings are cross-sectional, and therefore do not inform the direction of these relationships. However, they suggest that individuals who have used dating apps at least once throughout their lifetime also experience higher levels of DE. It is possible that individuals with greater body image concerns and eating disturbance prefer to interact with potential romantic partners on dating apps versus in real life, possibly due to increased control over their self-presentation [33]. Contrastingly, pressures surrounding physical appearance within dating app environments may trigger users to engage in DE behaviours in an attempt to enhance their perceived physical attractiveness, and thus, dating app success [30, 37, 41]. Future research is needed to disentangle the direction of these effects.

Our investigation into the association between DE and people’s motivations for using dating apps revealed that using dating apps for validation of self-worth or ease of communication was positively associated with DE. Negative beliefs regarding one’s self-worth are a central disturbance experienced by those with eating pathology; presumably, because DE behaviours reflect a maladaptive attempt to overcome states of low self-worth [11, 32, 40]. Moreover, it is plausible that individuals with greater eating pathology are motivated to use dating apps for their ease of communication, perhaps due to the interpersonal functioning problems (e.g., social skills deficits and social anxiety) associated with eating pathology [4]. For these individuals, dating apps may represent a more conducive environment for dating success than the offline world.

Using dating apps for the thrill of excitement and love negatively predicted DE. Characteristic of the thrill of excitement motive, sensation-seeking is a facet of impulsivity defined as the tendency to seek out novel and thrilling experiences [36, 45]. Sensation-seeking has been implicated in the development of DE behaviour, presumably, due to an urge to satisfy needs for risk and excitement through DE behaviours [21, 22, 44]. The finding that the thrill of excitement negatively predicted DE, may be attributable to our assessment of DE. We did not assess whether motivations for dating app use predicted specific dimensions of DE, such as dietary restraint or binge eating. This is important given that individuals who endorse high levels of sensation-seeking are more likely to report disinhibited ED behaviours such as binge eating and/or purging behaviours, whereas, those who report lower levels of sensation-seeking tend to engage in restrictive eating [21, 22, 44]. Our findings, therefore, suggest possible low sensation-seeking in individuals, which may highlight restrictive eating pathology. This aligns with past research linking low novelty seeking and a tendency to be hyper-protective when facing risk situations to DE and dietary restraint [2, 7].

We know of no conceptual framework to explain why love motivations might lead to lower eating pathology but note the finding as one warranting future replication and exploration. Together, the patterns of these motivation findings suggest that dating app users should not necessarily be considered a monolithic category. Our results highlight substantial heterogeneity among app users, and different motivational drives for using dating apps affecting DE outcomes.

### Clinical implications

Insofar as future research can replicate associations between motivations for dating app use and eating pathology, the present findings could be incorporated into practices that protect against the development of eating pathology in vulnerable individuals (i.e., those motivated to use dating apps for self-worth validation and ease of communication). For instance, dating app developers could partner with clinicians to implement a pre-screening instrument that assesses for interpersonal functioning deficits and the need for self-worth validation, and then notifies users about potential risks. Likewise, there is substantial value in clinicians’ enquiring about clients’ motivations for using dating apps, as these motivations could be used to provide insight into participants’ likelihood of DE. Thus, prompting more informed, healthful use, whilst helping these individuals feel more comfortable participating in online dating.

### Limitations

One limitation of the current study was that we did not assess the feedback people received from others while using the dating apps. Different predictions could be made for people who were frequently evaluated positively by others, versus those who were frequently rejected. Studies experimentally manipulating dating popularity [25], and any potential impacts on DE would further our understanding of these relationships. All variables in this study were assessed through self-report, thus, making it vulnerable to socially desirable responding and reliant on individual self-awareness. Likewise, we failed to differentiate between different subtypes of EDs. Future research should also seek to utilise designs that allow for investigation into the directionality of relationships using more complex statistical designs, both at the trait and state levels. Given documented disparities in BD and DE across sexual orientation groups [17] and the substantiated effects of sexual orientation on dating app use behaviour [38], comparisons between sexual orientation groups would be useful. Other demographic comparisons, including comparisons between individuals with different racial identities, would also be useful. Finally, our question about dating app usage asked participants if they had”ever” used dating apps could have been augmented by additional questions, such as questions asking about frequency and intensity of use, and perceived dating success.

### Conclusion

These findings extend current understandings of the relationship between dating app use and body image and DE by highlighting that dating app users are more likely to engage in DE behaviours than non-users. They provide novel insights into the varied motivations for dating app use and their unique impacts on DE. As dating app use continues to proliferate, enhancing our understanding of the mechanisms by which it may be harmful—and for which vulnerable groups—remains a salient area of research.

## Data Availability

The data included in the current study can be requested from the corresponding author on reasonable request.

## Abbreviations

BD: Body dissatisfaction
BMI: Body mass index
DE: Disordered eating
ED: Eating disorder
FNE: Fear of negative evaluation
